# Longitudinal Associations Between Parenting and Child Behaviour Problems and the Moderating Effect of Child Callous Unemotional Traits in Foster and Biological Families

**DOI:** 10.1007/s10578-022-01324-9

**Published:** 2022-03-03

**Authors:** Sabrina Schütte, Arnold Lohaus, Tabea Symanzik, Nina Heinrichs, Kerstin Konrad, Vanessa Reindl

**Affiliations:** 1 Developmental Psychology and Developmental Psychopathology, Department of Psychology, University of Bielefeld, P.O. Box 10 01 31, 33501 Bielefeld, Germany; 2 Department of Clinical Psychology & Psychotherapy, University of Bremen, Bremen, Germany; 3 Child Neuropsychology Section, Department of Child and Adolescent Psychiatry, Psychosomatics and Psychotherapy, Medical Faculty, RWTH Aachen University, Aachen, Germany; 4 JARA-Brain Institute II, Molecular Neuroscience and Neuroimaging, RWTH Aachen & Research Centre Juelich, Aachen, Germany

**Keywords:** Children in foster care, Parenting, Internalising and externalising problems, Callous-unemotional traits, Longitudinal study

## Abstract

The goals of the current study were to examine (i) differences in parenting between foster and biological parents, (ii) the longitudinal associations with children’s internalising and externalising problems and iii) the potential moderation of these by children’s callous-unemotional traits (CU traits). Data from 86 foster children (M = 4.44 years, male = 48%) and 148 biological children (M = 3.69 years, male = 49%) with their families were analysed in a longitudinal study with three measurement times. Parenting behaviour did not significantly differ between the foster and biological family groups. Significant longitudinal cross-lagged effects were found for parental warmth and support and children’s externalising problems. CU traits moderated the relationship between warmth and support and externalising problems of children. Findings suggest that parenting behaviours and child psychopathology do influence each other over time reciprocally and to a similar extent in both groups. However, there was also evidence for greater temporal stability of psychopathological symptoms and reduced responsivity to parental warmth in children with higher CU traits.

**Supplementary Information:**

The online version contains supplementary material available at 10.1007/s10578-022-01324-9.

## Introduction

Out-of-home care is considered an important intervention to ensure the security and adaptive development of infants and children when this is no longer possible in their biological family. Children living in foster care (CFC) are at increased risk for developing internalising (symptoms directed inwards, e.g., feelings of sadness) and externalising (symptoms directed outwards, e.g., aggressive behaviour) problems [1, 2]. Additionally, former maltreatment experiences of children may lead to long-term effects on functional and structural brain development [3], associated with behaviours that are adaptive in a maltreating family environment [4, 5], but that may cause problematic interactions in a foster family. Given the high risk of developing behavioural and emotional problems of CFC (e.g., [1]), the foster family care system may be crucial for the child’s development. Yet, a recent meta-analysis of longitudinal trajectories showed no changes in internalising and externalising behaviour problems and the child’s adaptive development in CFC [6], highlighting the importance of identifying protective and risk factors within the foster family care system to create optimal support for CFC.

Generally, many factors in (foster) families have been shown to influence children’s development, such as the socioeconomic status of a family [7], the number of maltreatment experiences in CFC [1], and the number of foster placement disruptions [8]. Furthermore, the (foster) parent’s parenting can play an important role in the development of children [9]. However, most research on the relations between parenting and child development have been conducted in the general population (e.g., [10, 11]). Therefore, these results may not be directly transferable to CFC. After entering foster care, behaviour problems may persist in spite of caregiving improvements because children have to cope with former maltreatment and/or neglect experiences in addition to adjusting to a new family and living environment [6]. Furthermore, given the unique history of CFC, different parenting strategies may be adaptive in CFC. Thus, the study at hand aims to examine whether the longitudinal associations between child behaviour problems and parenting differ between foster and biological families, and how parenting behaviour dimensions interact with children’s internalising and externalising problem behaviour in a longitudinal study. The results of this analysis may not only give hints regarding the potential relationships between parenting behaviours and child development, but also lead to tailored interventions for foster families to support the adaptive development of CFC.

Parenting behaviour is often categorised in responsiveness (or warmth), and parental demandingness [12], although also other categories exist (e.g., parental sensitivity; [13]). Parental demandingness can be further differentiated into behavioural control (which is often associated to positive child outcomes), harsh control (related to negative outcomes), and psychological pressure (attempts of manipulating the child, that are also linked to negative child outcomes; [12]).

With respect to *biological families,* the body of research examining the relationship between parenting behaviour and children’s behavioural problems is well-established. Meta-analyses of studies in the general population showed small to moderate associations between dysfunctional parenting (i.e. overly strict/unclear and inconsistent parental behaviour or forms of psychological pressure) and an increased risk for depression and anxiety in children [11, 14, 15], small negative associations between functional parenting (i.e. provision of warmth/appropriate demanding and control) and externalising problems in children [10, 16], as well as moderate positive associations between dysfunctional parenting dimensions and externalising behaviour [16]. Also, previous research further showed that these associations seem to be rather reciprocal in longitudinal studies (e.g., [16]), i.e., studies showed that certain parenting behaviours may on the one hand evoke compatible child developmental pathways. On the other hand, child behaviours also may evoke certain parental reactions and behaviours.

Research in *foster families* regarding the parenting and development of CFC is not as elaborated as in biological families. Vanderfaeillie et al. [17] showed in their longitudinal study that problem behaviours in CFC were significantly associated with the use of negative parenting in foster mothers. Moreover, Chodura et al. [18] found in their meta-analysis similar associations between parenting and child development in foster families, as had been previously found in biological families; however, most of the studies (34 out of 43) were cross-sectional. Additionally, Orme and Buehler [7] concluded that approximately 15 percent of foster parents manifest potentially poor or troubled parenting as reported by youth welfare workers (however, of note is that comparable studies in biological families are missing). On the one hand, this is important because of the high vulnerability of CFC [8]. Because CFC have experienced more adverse life events in early childhood, they may need a more supportive environment for their adaptive development, for instance provided through functional parental behaviours. On the other hand, poor parenting may also occur because of more problematic behaviours shown by CFC, as indicated in longitudinal studies (e.g., [17]).

Directly compared, differences have been found in the parenting behaviour of foster and biological parents, but the evidence for the directions of the differences is mixed and limited. Fisher et al. [19] found that foster parents showed more dysfunctional parenting behaviour compared to a general population sample of parents. In contrast, Lucey et al. [20] found that foster parents reported less verbal and corporal punishment than biological parents. In this study, differences in parenting behaviour were explained by demographic differences between the two groups: foster mothers were found to be significantly older and married more often than biological mothers [20]. Furthermore, Linares et al. [21] found that foster parents reported more clear expectations (regarding rules about family routines) than biological parents *despite* demographical differences. Additionally, Ehrenberg et al. ([22]; using the same sample as in this study) analysed different risk factors for the development of CFC. They found differences in parenting behaviour and (foster) parents’ stress between foster care and biological families that were generally explained by child problems. Furthermore, they assumed interactional processes between parenting variables and child problems, which they did not investigate further because of a different focus of their analysis. Taken together, existing studies often examined longitudinal associations between parenting behaviour and child problems only in families with CFC, however, without comparing the results to biological families. Further, when comparing the two groups, such comparisons were mostly done for only one aspect, either parenting behaviour or child problems.

To summarise, the evidence found in biological families may not be transferable to foster families. There may be differences between both groups due to differences in demographical characteristics between biological and foster families (e.g., the socioeconomic status and age of the parents, [23]), differences in children’s characteristics and behaviour, and possibly also due to differences in parenting behaviour. There is, however, currently a large research desideratum in identifying longitudinal relationships between parenting behaviour in foster and biological families and children’s internalising and externalising problem behaviour. To our knowledge, no former study used cross-lagged panel models to compare the relationships between parenting behaviours and children’s internalising and externalising problem behaviours in biological *and* foster families. Therefore, it remains unclear as to whether parenting more strongly influences child development or vice versa, in particular in foster families.

Considering a well-examined theoretical model, Belsky’s process model of parenting [24] may help us to understand such interaction effects. According to this model, parenting does have an influence on child development (e.g., child problem behaviour), but is influenced (among other context-related components) by child characteristics (e.g., temperament). Previous studies revealed that callous-unemotional (CU) traits as child characteristics are potentially important moderators of the link between parent–child relationships and developmental outcomes, in particular with respect to externalising behaviours [25]. Children scoring high on CU traits show a lack of empathy and guilt, emotional ‘coldness’, and disregard for feelings of others together with physiological hypoarousal and reduced responsivity to punishment [26]. As such, CU traits have been demonstrated as a serious risk factor for externalising behaviour problems and for a particularly persistent course of conduct disorder [27]. Emerging research suggests that high CU traits can be explained by two different variants [28]. Primary variants of CU traits are thought to emerge from genetic factors and are associated with insufficient arousal to emotional cues. Secondary variants are supposed to emerge from trauma experiences, leading to emotional numbing, avoidance and inhibition of empathy as reaction on traumatic environments [28]. While earlier studies propose that the problem behaviours in children with high levels of CU traits become rather irresponsive to parenting—in line with primary CU variants [29, 30]—more recent evidence suggests the opposite: parental warmth in clinically-referred, conduct-disordered children with elevated CU traits led to a decrease in the risk for antisocial outcomes [31]. Further, in non-clinical samples, more positive dyadic parent–child relationships predicted a decrease in future behaviour problems only in children with high, but not in children with relatively low CU traits [32]. This may imply that parental warmth is a dimension of special interest for CFC, assuming they have on average higher levels of CU traits due to experiencing more adverse life events that may lead to the emergence of higher secondary CU traits. Interestingly, a recent adoption study found that low parental warmth displayed by adoptive parents disrupted the heritability of CU traits [33, 34]; furthermore, a recent twin study suggested that parenting is related to child CU traits and aggression, over and above genetically-mediated effects, with low parental warmth being a unique correlate of CU traits [35]. However, neither child temperament nor other child characteristics per se shape parenting, but rather the ‘goodness-of-fit ‘ between parent and child might determine the development of parent–child relationships and the developmental outcomes in the child.

### Research Questions and Hypotheses

Taking into account the potential demographic differences between samples, the goals of the current study were to investigate (i) whether foster and biological parents differ in their parenting behaviour, (ii) whether longitudinal pathways differ between foster families and biological families and how parenting behaviour and internalising and externalising problems of children are longitudinally related, and (iii) whether the relationship between parenting behaviour and internalising and externalising is moderated by CU traits.

Based on the research findings outlined above as well as Belsky’s process model of parenting [24], direct longitudinal relationships are expected between parenting behaviours and children’s problem behaviours in both, foster and biological families, although the literature is inconsistent as described above. That is, negative associations are expected between functional parenting and low internalizing and externalizing child behaviour, and vice versa for dysfunctional parenting and child mental health. Across groups, a moderating effect of children’s CU traits is expected for the association between parental warmth and support and children’s externalising problems based on previous findings. Considering the existing literature, we could expect both: a stronger association between parenting and externalising problems in the group of children with high CU traits (e.g., [32]) or a stronger association in the low CU trait group (e.g., [30]).

## Methods

### Sample

Data were collected as part of a larger project investigating the development of children in foster care (GROW&TREAT project; for a complete list of measurements see: https://www.uni-bremen.de/klips/forschung/abgeschlossene-forschungsprojekte/grow-treat). The longitudinal data collection included three assessment waves (T1, T2, T3), with approximately six-month intervals between the assessments. After T1, a parent training program was offered to approximately half of the foster families. Since no effects of the intervention were found on any of the outcome variables [36], or for the variables used in the study at hand, this was not considered further in the analyses.

CFC were recruited via youth welfare offices in three German regions. Youth welfare officers were asked to address non-kinship care foster families with children aged between two and seven years, who lived for no more than 24 months in the current foster family and had experienced maltreatment and/or neglect before being placed in foster care. Biological families in the same age range were recruited via postings or parents’ evenings in kindergartens, schools and paediatricians’ offices. The person(s) holding custody of the (foster) child provided written informed consent for the child’s participation, and in the case of (foster) parents for their own participation. Families received an incentive of 30€ for each assessment and an additional 30€ after the final assessment. Ethical approval was obtained by the German Society for Psychology. For a detailed description of the recruiting process, see Chodura et al. [23].

A total of N = 94 CFC and N = 157 children in biological families (CBF) participated in the study with their (foster) parents. Of these children, eleven pairs and one trio were biological siblings. In the case of siblings, one child per family was randomly selected to avoid sample biases. This resulted in a final sample size of N = 86 CFC and N = 148 CBF at T1. Fifteen families dropped out of the study after T1 (6 CFC, 9 CBF) and six families (4 CFC, 2 CBF) after T2. Descriptive results for the two groups are provided in Table 1. Groups significantly differed in terms of child’s age, parents’ age and children’s CU Traits.

**Table 1:** Sample description and comparison between foster and biological families in sociodemographic variables

Variable	Group	N	M	SD	T	df	p-value
Age of children (in months), T1	CBF	148	53.28	17.36	3.76	232	< .001
	CFC	86	44.30	18.00			
Age of mothers	CBF	148	35.44	5.31	− 6.40	144.73	< .001
	CFC	86	40.93	6.84			
Age of fathers	CBF	145	38.54	5.89	6.81	223	< .001
	CFC	80	44.49	6.92			
Socioeconomic status of families	CBF	130	14.88	4.03	− 0.78	202	.439
	CFC	74	15.32	3.63			
CU Traits	CBF	148	.79	.24	− 4.79	129	< .001
	CFC	86	1.01	.40			

	Group	N	Percentage (Male)		χ^2^	df	
Gender of children	CBF	148	47.97		.02	1	.899
	CFC	86	48.84				

*CBF* = children in biological families, *CFC* = children in foster families. Age is reported in years. *SES* = socio-economic status

### Instruments

Variables were measured via questionnaires provided to the (foster) parents. Parenting scales were asked to be filled out by the primary caretaker of the child, which was mostly the (foster) mother. Questions regarding the child were also mostly filled out by the main caretaker (70.04% mothers; 6.82% fathers), and in a few cases by both parents (7.38%).

*Parenting* was measured by the Zurich Brief Questionnaire for the Assessment of Parental Behaviours (ZBQ; [37]). The ZBQ was originally designed as a child-report instrument to assess parental behaviour from the children’s perspective, but because of the young age of the sample was employed as a caregiver-report in the current study. The ZBQ consists of 27 items, assigned to three scales: *warmth and support* (12 Items, e.g., ‘I praise my child, when he or she does something good.’), *psychological pressure* (9 Items, e.g., ‘I think my child is ungrateful, when he or she disobeys.’), and *demands and control* (6 Items, e.g.,‘I expect my child to keep his or her things in order.’). Thus, the ZBQ divides the commonly used category of demandingness into two subcategories related to appropriate control and psychological pressure. Items were answered on a four-point Likert-scale ranging from (1) ‘not true’ to (4) ‘very true’. For each scale, items were averaged. Since the scale *demands and control* includes many items that do not fit to the young age of the children in the current sample (e.g., ‘I always want my child to ask me before he or she leaves the house.’), it was excluded from the current analysis. Additionally, using only the two scales *warmth and support* and *psychological pressure* does fit better to the common categories of responsiveness and demandingness [12] as described above. The internal consistencies, measured by Cronbach’s alpha, were satisfactory for *warmth and support* (α from 0.71 to 0.72), but rather low for *psychological pressure* (α from 0.52 to 0.59).

*Child emotional and behaviour problems* were assessed using the German versions of the Child Behaviour Checklist (CBCL). The CBCL 1½-5 [38] was employed for children aged 2 to 4 years and the CBCL 4–18 [39] for children aged 5 years and older. *T*-scores (for demographical analyses) and *z*-scores (for the analyses of the research questions) of the *internalising and externalising problem behaviour* scales were used in the analyses below. Internal consistencies were satisfactory to high for *internalising problem behaviour* (α from 0.75 to 0.90) and excellent for *externalising problem behaviour* (α from 0.86 to 0.92).

*Children’s callous-unemotional traits* were examined using the 24-item Inventory of Callous-Unemotional Traits (ICU; [40, 41]) at T1. The ICU is available as a school and preschool version. The versions are identical except for one item (school version: ‘Is concerned about schoolwork.’ vs. preschool version: ‘Seems motivated to do his/her best in structured activities.’). Because both preschool and school-aged children participated in this research project, the school version was applied. For the current analysis, the different item of the two versions was removed, resulting in a 23-item ICU version. Internal consistencies were the same for the original and the reduced ICU scales (*α* = 0.58). A mean score was calculated and values were *z*-standardised for further analyses.

The family’s *socio-economic status (SES)* was assessed based on the social class index by Winkler and Stolzenberg [42]. Families reported on their income, mother’s and father’s employment status, school and professional education. Following the instructions of the manual [42], an overall score for the family’s SES was derived (possible range 3–21).

### Statistical Analysis

Statistical analyses were conducted in *R* (Version 4.0.3; [43]) and *SPSS 26* [44]. All tests were two-tailed and *p*-values below 0.05 were considered statistically significant.

#### Multiple Imputation

The overall missing rate was 8.37% for the ZBQ and CBCL and can therefore be interpreted as reasonable [45]. Missing value analysis was done in SPSS including Little’s MCAR test, which indicated that the examined variables were missing at random. To enhance the power of the models, data sets were imputed on the scale level [46]. Multiple imputation was done with the R package ‘mice’ [47] with 10 iterations [48]. Afterwards the imputed data sets were merged into a single data set by computing the mean of all imputed values using the R package ‘sjmisc’ [49].

#### Research Questions

To examine whether parenting behaviour differed between foster and biological parents, a series of linear mixed models was calculated using the R package ‘lme4’ [50]. Linear mixed models expand on the ordinary linear regression model by including both fixed- and random-effect terms. Thereby, this allows to incorporate the lack of independence in longitudinal data sets [51]. Following the recommendations by Luke [52], final models were fitted by REML, and *p*-values were derived using the summary function of the R package ‘lmerTest’ [53] with the Satterthwaite approximation for the degrees of freedom. First, to test group differences in parenting, two linear mixed models were calculated with *warmth and support* and *psychological pressure* as the dependent variable, respectively. Models included a random intercept for subject and the fixed effects of time, group (0 = CBF, 1 = CFC) as well as of relevant covariates: child’s age (at T1, in months), mother’s age (at T1, in years) and SES (at T1). Time was dummy coded with T1 as a baseline category. The age of the (foster) mother and (foster) father was highly correlated (*r* = 0.74, *p* < 0.001), which may result in issues of multicollinearity. As (foster) mothers mostly filled out the questionnaires, only the age of the mother was included in the models. Second, to test whether the effect of the measurement time point differed between groups, the time x group interactions were added to the linear mixed models described above.

**Fig. 1:**
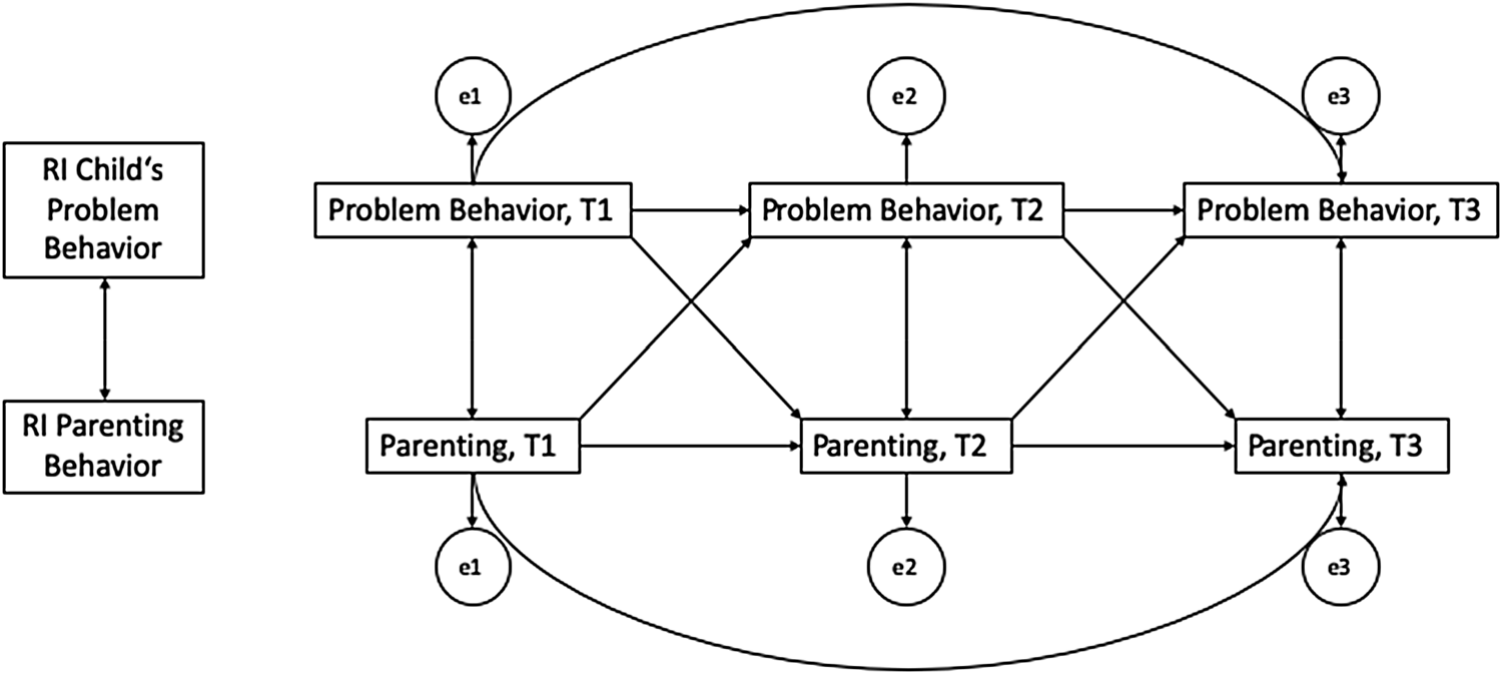
Theoretical RI-CLPM between Parenting and Children’s Problems

To examine the longitudinal pathways between parenting and children’s development, random intercept cross-lagged panel models (RI-CLPM; [54, 55]) were calculated, similar to Lohaus et al. [56], who computed cross-lagged panel models (CLPMs) to analyse the longitudinal pathways between parents’ stress and child problems in foster and biological families (using the same data set). CLPMs calculate both cross-lagged effects, i.e., longitudinal effects between two variables, and autoregressive effects, i.e., longitudinal effects of a variable on itself. Therefore, autoregressive effects can indicate longitudinal stabilities of variables. Preliminary analyses which showed high longitudinal stabilities of the CBCL (*r* between 0.44 and 0.84) and the ZBQ scales (*r* between 0.37 and 0.70, Supplementary Table 1) motivated our decision to calculate RI-CLPMs instead of the traditional CLPMs. In contrast to the CLPM, the RI-CLPM accounts for stable, rather time-invariant differences between individuals by including random intercepts. Thus, it decomposes longitudinal data into stable, between-person differences (here, differences in parenting and internalising/externalising symptoms) and into temporal, within-person dynamics. In fact, simulations have shown that CLPMs can lead to spurious results if the stability of the constructs is to some extent trait-like [54]. The RI-CLPMs were calculated in the R package *lavaan* [57]. Six RI-CLPMs were formulated for the relationships between parenting (ZBQ scales: warmth and support/psychological pressure) and child’s development (CBCL scales: internalising/externalising problems) with group (0 = CBF, 1 = CFC) as a grouping variable (see Fig. 1 for a graphical representation of the models). For each RI-CLPM, two models were compared via chi-squared difference tests: one model in which there were no constraints across the groups and one model in which the lagged regression coefficients were constrained to be identical across the groups [55]. The model fit was evaluated using the root mean squared error of approximation (RMSEA) value and χ^2^-test results [58]. Hereby, RMSEAs smaller than 0.05 indicate a good, smaller than 0.08 an adequate, and smaller than 0.10 an acceptable model fit [58]. Because the child’s age differed between groups, age was partialled out in the CBCL and ZBQ scales. We calculated separate models for each scale of parenting behaviour because of better interpretability of the results (e.g., *psychological pressure* is presumed to have a negative effect on child emotional and behaviour problems, while *warmth and support* is expected to have a positive effect).

For the moderation analyses of CU traits on the relationship between parenting behaviour and children’s emotional and problem behaviour, the variables of parenting behaviour and internalising and externalising problems were averaged over the three assessment points. Prior analyses confirmed that the mean CBCL/ZBQ values did not differ between assessment points, as indicated by a series of *t*-tests (all *p values* > 0.159). To test for moderation effects, six multiple regression models were calculated, one for each parenting-child behaviour relationship. Averaged child internalising and externalising problems were defined as the dependent variable (as done by [32]); group, parenting behaviour, CU traits and their interactive effects were included as predictors. Interaction effects were included by multiplying the values of the respective variables. Since we found no significant three-way interactions, the final models included the main effect of group, as a control variable, in addition to the effects of parenting behaviour, CU traits and their two-way interaction.

## Results

Table 2 shows the descriptive statistics for child problems and parenting behaviour scales for both sample groups. Internalising and externalising child behaviours and CU traits were significantly higher for CFC than for CBF, except for internalising problems at T2 (*T*  = − 1.77, *p* = 0.078). Furthermore, CFC showed significantly more CU traits (*z*-values: *M* = 0.43, *SD* = 1.22) than CBF (*M* = − 0.25, *SD* = 0.74; *T* = − 4.75, *p* < 0.001).

**Table 2:** Descriptive statistics of the study variables

	T1		T2		T3	
	CBF	CFC	CBF	CFC	CBF	CFC
Externalizing problems (*M*, *SE*)	50.72 (.78)	54.90 (1.16)	48.51 (.77)	53.58 (1.28)	47.50 (.78)	53.82 (1.27)
Internalizing problems (*M*, *SE*)	50.50 (.75)	54.07 (1.20)	47.67 (.65)	50.08 (1.33)	46.16 (.74)	50.49 (1.26)
CU traits (*M*, *SE*)	.79 (.24)	1.01 (.40)	–	–	–	–
Warmth and support (*M*, *SE*)	3.72 (.02)	3.67 (.03)	3.70 (.02)	3.62 (.03)	3.73 (.02)	3.70 (.03)
Psychological pressure (*M*, *SE*)	1.74 (.03)	1.72 (.03)	1.75 (.03)	1.71 (.04)	1.63 (.03)	1.66 (.04)

*CBF* = children in biological families (*N* = 148), *CFC* = children in foster families (*N* = 86); values accounted for imputed data

### Differences in Parenting Behaviour

The results for the first research question regarding potential differences in parenting behaviours between foster and biological parents showed that the effect of group, the effect of time, and the interaction effect of group and time were not significant for both parenting behaviour scales (Table 3). Thus, across assessment points, foster and biological parents did not significantly differ in parenting behaviour. For both groups, the scale *warmth and support* showed higher means (3.62–3.73), whereas the means for *psychological pressure* were rather low (1.63–1.75). Table 3 also shows only for psychological pressure a significant effect of child’s age (b = − 0.01**), but not for the other covariates mother’s age and SES, and no significant effect of these variables on warmth and support.

**Table 3:** Linear mixed models predicting parenting variables from group, time and relevant covariates

	Warmth and support	Psychological pressure
Fixed effect		
Intercept	.04 (.34)	1.10 (.35)**
Child’s age	< .01 (< .01)	− .01 (< .01)**
Mother’s age	< − .01 (< .01)	− .02 (.01)
SES	< .01 (.01)	.01 (.01)
Group	− .20 (.15)	− .03 (.15)
Time T2	.05 (.08)	.08 (.08)
Time T3	− .02 (.08)	− .02 (.08)
Group by Time T2	− .10 (13)	− .11 (.13)
Group by Time T3	.09 (.13)	.12 (.13)
Random part		
Intercept	.45 (.67)	.49 (.70)
Residual	.46 (.68)	.43 (.65)

For the fixed effects, estimates are presented with the standard errors in parenthesis. For the random part, variances are presented with the standard deviations in parenthesis. Child’s age = child’s age at T1 in months. Mother’s age = mother’s age at T1 in years. SES = family’s socio-economic status at T1 (centered). Measurement time point was dummy coded with T1 as the baseline category (Time T2: 0 = T1, 1 = T2; Time T3: 0 = T1, 1 = T3). Group: 0 = CBF, 1 = CFC. *p < .05, ** *p* < .01, *** *p* < .001

### Longitudinal Pathways Between Parenting Behaviour and Children’s Development

For Research Question 2, which analyses the reciprocal longitudinal effects of parenting behaviour and children’s problem behaviour, RI-CLPMs were computed for each combination of parenting and children’s problem behaviour scales. Supplementary Tables 2 and 3 show the intercorrelations for the problem behaviour and parenting behaviour scales for the CFC and the CBF group, respectively. For each combination, the unconstrained, multiple-group version of the RI-CLPM was compared with a model in which the lagged effects were constraint to be identical across groups. The chi-squared difference tests were not significant, indicating that imposing the equality constraints on the lagged parameters across groups was tenable. Thus, the relationships between parenting and child internalising/externalising problems were not significantly moderated by group (biological vs. foster families).

**Fig. 2:**
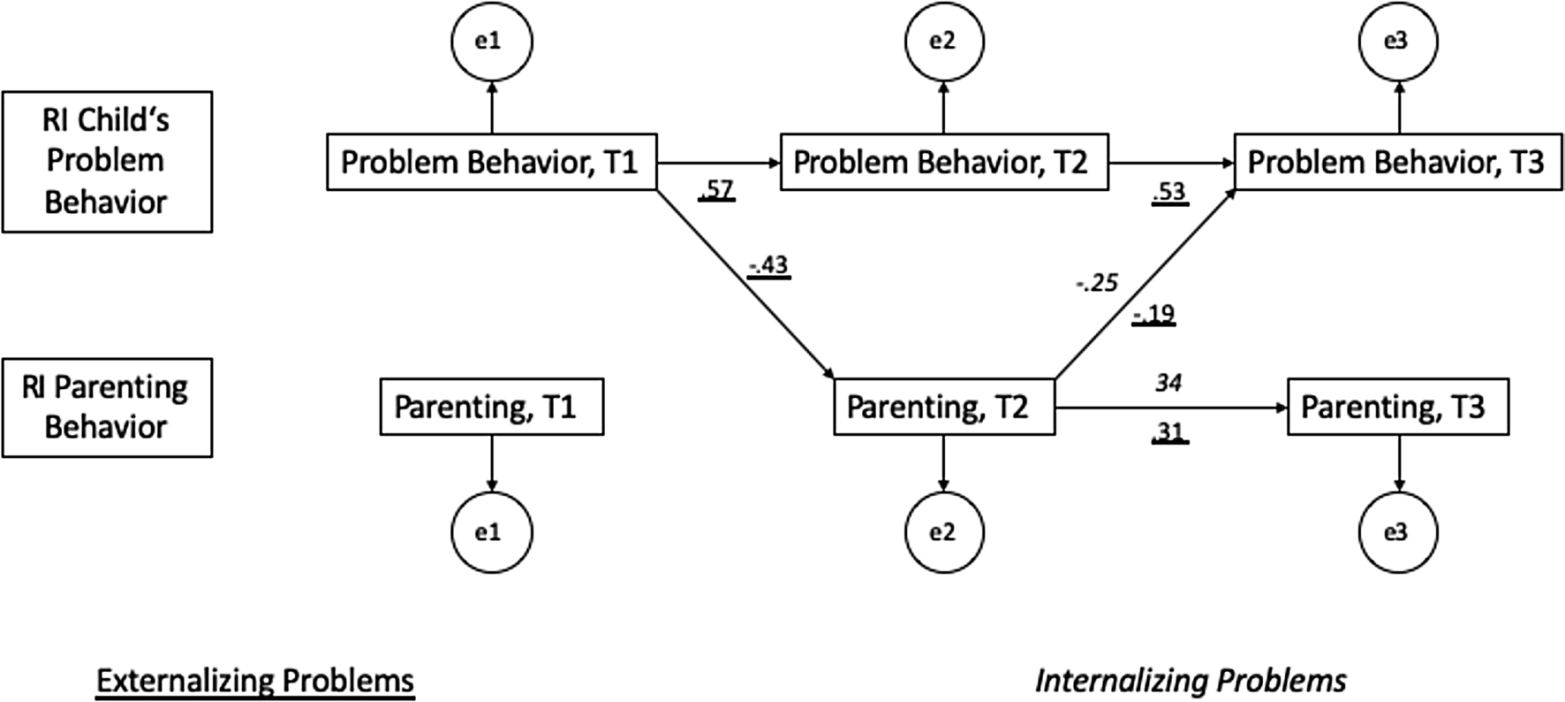
RI-CLPM between warmth and support and children’s problems (Externalizing: *χ*^*2*^ (15) = 77.64, *p* < .001, *RMSEA* = .03; Internalizing: *χ*^*2*^(15) = 538.18, *p* < .001, *RMSEA* < .01). Displayed are standardized values of the significant paths

**Fig. 3:**
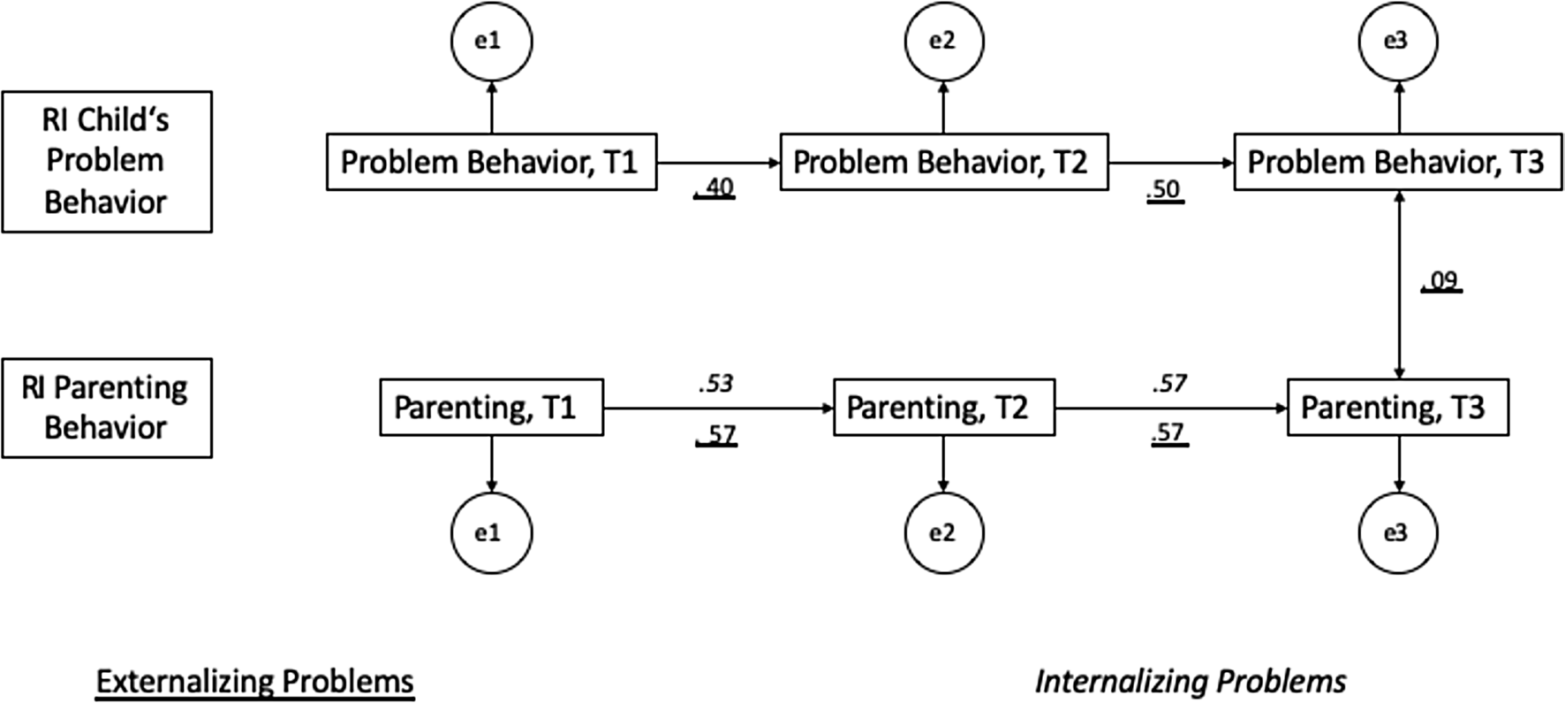
RI-CLPM between psychological pressure and children’s problems (Externalizing: *χ*^*2*^(15) = 832.49, *p* < .001, *RMSEA* = .01; Internalizing: *χ*^*2*^(15) = 608.38, *p* < .001, *RMSEA* = .08). Displayed are standardized values of the significant paths

In the following, the constrained models are reported. Overall, the model fits were acceptable to good (see Figs. 2, 3). At the between-person level, no associations were found between children’s behaviour problems and parenting.

*Autoregressive paths* of children’s internalising problems were not significant in any model; in contrast, all autoregressive paths for children’s externalising problems were significant (Figs. 2, 3). Regarding parenting behaviour, the autoregressive path of warmth and support at T2–T3 (with externalising: *b* = 0.31, *p* = 0.008, with internalising: *b* = 0.34, *p* = 0.002, Fig. 2) and both autoregressive paths of psychological pressure (T1–T2: *b* = 0.57, *p* < 0.001; T2–T3: *b* = 0.57, *p* < 0.001, Fig. 3) were significant.

Significant *cross-lagged paths* were found from externalising problems at T1 to warmth and support at T2 (*b* = − 0.43, *p* = 0.001), and conversely, from warmth and support at T2 to externalising problems at T3 (*b* = − 0.19, *p* = 0.018), indicating that more externalising problems at T1 were associated with less warmth and support at T2, and more warmth and support at T2 was associated with less externalising problems at T3. Additionally, the cross-lagged path from warmth and support at T2 to internalising problems at T3 (*b* = − 0.25, *p* = 0.015) was significant, i.e., more warmth and support at T2 was associated with less internalising problems at T3.

For psychological pressure, no significant cross-lagged paths were found (neither with internalising nor with externalising problems), only externalising problems and psychological pressure were positively associated at T3 (*b* = 0.09, *p* = 0.003).

### Moderation Effects of CU Traits

**Fig. 4:**
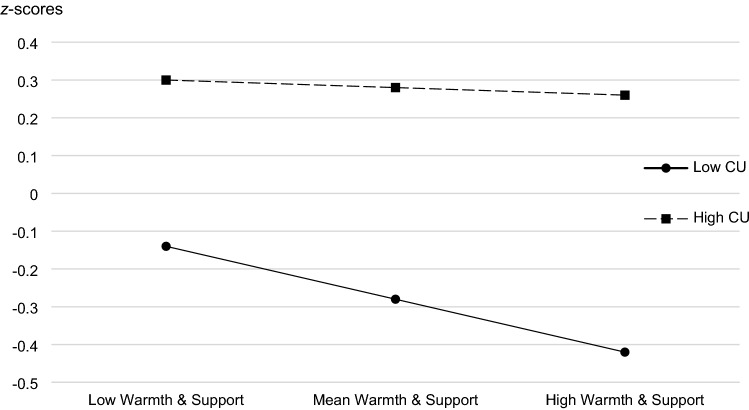
Callous-unemotional traits moderate the effect of warmth and support in parents on children’s externalizing problems. *CU* = CU Traits. Low Warmth & Support = 1 SD below the mean. Mean Warmth & Support = mean. High Warmth & Support = 1 SD above mean. Low CU = 1 SD below Mean. High CU = 1 SD above mean. Group was included as a covariate in the multiple regression models (not depicted)

Regarding Research Question 3, examining the potential moderation effect of CU traits on the relationship between parenting behaviour and internalising and externalising problems in children, significant main effects were found for warmth and support (*b* = − 0.14, *p* = 0.025), CU traits (*b* = 0.28, *p* < 0.001), and group (*b* = 0.27, *p* = 0.014) in addition to a significant interaction effect between warmth and support and CU traits (*b* = − 0.13, *p* = 0.035) on externalising problems (*R*^*2*^ = 0.21, *F* = 17.98, *p* < 0.001). The simple slope results are displayed in Fig. 4. For children with low levels of CU traits (1 SD below mean of the sample), externalising problems decreased with higher levels of warmth and support. For children with high levels of CU traits (1 SD above mean), the slope was close to zero, indicating that no effect of warmth and support on children’s externalising problems was found. For the remaining parenting-child behaviour relationships (including children’s internalising problems), no significant interaction effects were observed, indicating only additive effects of psychological pressure and CU traits on children’s internalising and externalising problems, respectively (see Supplementary Tables 4 and 5 for the results of the regression analyses).

## Discussion

The aim of the study at hand was to examine the longitudinal associations between parenting behaviour and children’s emotional and behavioural problems in biological and foster families and the potential influence of children’s CU traits. In summary, no differences were found between the CFC and the CBF groups in parenting behaviours and associations with children’s internalising and externalising symptoms. Independent of the group (CFC versus CBF), the associations showed cross-lagged longitudinal effects between warmth and support and internalising and externalising problems.

The first research question examining if parenting behaviour is different between the two sample groups showed no significant differences in parenting behaviour for both parenting scales. In general, foster parents seem to show the same parenting behaviours aiming to reduce children’s behaviour problems as biological parents. Therefore, the results complement not only previous research in biological families (e.g., [14–16]), but also the rather small body of research on parenting in foster families [18].

Regarding the second research question examining the longitudinal pathways between parenting behaviour and children’s internalising and externalising behaviour, random intercept cross-lagged panel models were computed. Overall, χ^2^-tests indicated that it is reasonable to assume identical longitudinal cross-lagged effects between the two groups. In summary, our results showed a high longitudinal stability for psychological pressure and no cross-lagged effects with children’s internalising/externalising symptoms. In contrast, for parental warmth and support, several cross-lagged effects were found, in particular for child’s externalising problems. Specifically, more externalising problems at T1 predicted less warmth and support at T2 and more warmth and support at T2 predicted less externalising *and* internalising problems at T3. These negative longitudinal associations have also been found in previous research examining the emotional and behaviour problems of CFC (e.g., [13]) and CBF (e.g., [16]), indicating a rather reliable finding. In conclusion, in particular the provision of warmth and support was shown to have an influence on child behaviour and emotional problems when both parenting dimensions were investigated separately. Associations may have differed had we included both parenting dimensions simultaneously into the model. It is therefore unclear if both contribute individually above and beyond the other or if their shared variance (i.e., construct overlap) is more responsible for these results.

The fact that more autoregressive effects were found for externalising than for internalising behaviours may be explained by the poorer observability of internalising problems in general (e.g., [59]). The present study can provide early insights into the different association dynamics between internalising and externalising problems and parenting behaviours and thus help to fill the research desideratum discussed above and to better understand the previous mixed evidence for foster families [19, 20].

The effect sizes were generally rather low between parenting and child emotional and behaviour problems. This also had been found in a meta-analysis and literature review by Chodura et al. [18], which showed that externalising and internalising problems were associated with parenting behaviours to a low to moderate degree. Additionally, the authors found generally few other variables in families that were associated with internalising and externalising problems in CFC, such as the length of stay in the foster family, possibly indicating that previous experiences prior to entering foster care may have a larger effect. This goes along with Goemans et al. [6], who also found few variables influencing the trajectories of child development in CFC.

Furthermore, CFC showed significantly more emotional and behaviour problems than CBF in the study at hand, and additionally the longitudinal stabilities of internalising and externalising problems were significantly higher in CFC than in CBF (see also [56, 60], using the same sample as the current article). This may explain some of the low cross-lagged effects in foster families. However, no differences were found between both groups regarding these longitudinal associations. That is, foster parents may show the same parenting behaviours as biological parents, indicating appropriate parenting responses, even though CFC show more and longer lasting problem behaviours. Foster parents still are seen as the most important target group for interventions to support the development of CFC, possibly due to the fact that there may be the most potential for change. This is also in line with cross-sectional and intervention studies that indicate that parenting behaviour and the parent–child relationship in foster families may positively influence the child’s stress response systems, such as the hypothalamic-pituitary-adrenocortical (HPA) axis and may thereby mitigate the possible consequences of early life stress ([61, 62], using the same sample as the current article). Alterations in the HPA axis may constitute a risk factor the development of later mental and somatic health problems [63]. Additionally, there may be adjustment processes that can be seen only in examinations longer than one year, i.e., rather long-term effects. Consequently, (foster) parents and youth welfare workers should not neglect the potential effects on children’s problem behaviour, even though they may be small.

The third research question investigated whether CU traits moderated the associations between parenting behaviour and children’s emotional and behaviour problems as a potential factor which could explain additional variance of these associations. In line with earlier studies, our findings suggest that CU traits indeed moderated the relationship between parental warmth and support and children’s externalising behaviour [29, 30]. This effect, again, supports Belsky’s model, indicating that child characteristics may be important moderators of the link between parent–child relationships and developmental outcomes and explains further the longitudinal effects observed in the current study. This is, CU traits as child factors may set directions for parenting behaviour: children with high CU traits may show difficult behaviour resulting in lower associations between parental warmth and support and children’s externalising problems. However, in contrast to some previous studies in non-foster care families [31, 32], this effect was driven by a higher association between positive parenting behaviour and the child’s externalising behaviour present only in those children scoring low in CU traits (no interaction effect was found with group, indicating no differences in this moderating effect for foster and biological parents). This finding is in line with a recent study showing that positive parenting was most relevant when distinguishing between youth with conduct disorder and high versus low levels of CU traits using a machine learning approach [64] and with intervention studies showing lower effect sizes in subjects with higher CU traits. While our assessment methods differed from those of Kochanska et al. [32], shared positive affect is most closely related to the ZBQ subscale *warmth and support*. In line thereof, we found no moderation effects for the ZBQ scale *psychological pressure*, and no moderation effects for internalising problems. This further supports our conclusion that in particular the provision of warmth and support has an important influence on the children’s behaviour problems. Note, however, that in the present study children’s levels of CU traits and parent’s levels of psychological pressure were generally low (CU traits: *SD* = 0.24 for CBF and *SD* = 0.40 for CFC), which might have influenced our findings. In summary, future research is needed to further examine the influence of early adverse life events, elevated CU traits, and parental warmth and support, on children’s development.

### Limitations and Recommendations for Future Research

The present study used questionnaires filled in by mostly one (foster) parent. Possible effects may therefore be traceable to an effect of the same source. Yet, this is unlikely given the overall small associations between parenting and child problems. Further, Chodura et al. [18] generally found low to medium effect sizes in their meta-analysis, considering all types of studies. Nevertheless, to further support the observed effects, future research should employ a multi-informant, multi-method approach in measuring parenting and children’s problem behaviour (e.g., including behavioural observations, reports from teachers, both parent reports). These operationalisations could give deeper insights into parents’ and children’s behaviour but may also be associated with reduced participation rates of populations [65].

Regarding the operationalization of parenting, the internal consistencies of the parenting scale psychological pressure also should be taken into account. The low internal consistencies may have influenced the cross-sectional and longitudinal associations with child problems. One explanation could be the age of children in the sample groups. Internal consistencies of the ZBQ were higher in the validation studies [37] with *α* between 0.73 and 0.75 and should therefore be interpreted with caution.

Possible self-selection effects which may lead to more homogeneous samples should not be overlooked [66]. The present study tried to reduce this effect by contacting case workers of youth welfare institutions and asking them to provide the study information to as many foster families as possible. Additionally, the CBF group was recruited in similar regions to reduce differences in demographic characteristics, which may have increased homogeneity. The limited generalizability also should be noted in terms of the results regarding CU traits. CFC are a group of children with certain adverse experiences and the results, therefore, are not simply applicable to other population groups.

According to Éthier et al. [1], victims of chronic maltreatment show more emotional problems, aggressive behaviour and social withdrawal problems than victims of transitory maltreatment. The chronicity and severity of maltreatment and neglect experiences of CFC groups therefore should be considered in future research. It might be helpful to distinguish between maltreated children with and without clinical symptoms of psychiatric disorders, which would have exceeded the statistical power in the present sample. Éthier et al. [1] also pointed out that children’s developmental improvement in the transitory maltreatment group emerged not directly after the end of maltreatment, but rather gradually. This may show that chronically maltreated children need more time to show improvements in development after the transition into a non-maltreating environment [1], which is important information for youth welfare workers as well as for foster parents and may give better insights into differential developmental pathways in CFC.

## Conclusion

The study at hand was the first to examine parenting behaviour and children’s problem behaviour in foster and biological families with cross-lagged panel analyses. Complementary to many former studies, and in line with Belsky’s process model [24] of parenting, we could show some longitudinal cross-lagged effects between parenting’s warmth and support and children’s problem behaviour, in particular for externalising problems. This gives an important insight to reduce behavioural problems with tailored parenting behaviours, especially for children with maltreatment experiences. Additionally, regardless of being a foster or biological parent, children’s behaviour may be challenging and it is important to ensure proper support for parents to minimize long term negative effects of aversive parental behaviour on children’s development. Since foster parents may be confronted with more challenges and stressors, these should be carefully considered in foster care programs.

## Summary

Former maltreatment experiences of children in foster care (CFC) may lead to emotional and behaviour problems that need tailored parenting behaviours of foster parents. Former research does, however, leave a gap regarding the longitudinal associations of parenting behaviour and child’s internalising and externalising problems in foster families, and whether these differ from the associations in biological families. families are poorly understood, and it is unclear whether these differ from biological families. Thus, the goals of the current study were to examine i) differences in parenting between foster and biological parents, ii) the longitudinal associations with children’s internalising and externalising problems and iii) the potential moderation of these by the children’s callous-unemotional traits (CU traits). Data from 86 foster families and 148 biological families (mean age = 4.16 years) were analysed in a longitudinal study with three measurement times, approximately six months apart. Parenting was assessed via self-report on the *warmth and support* and *psychological pressure* scales of the Zurich Brief Questionnaire for the Assessment of Parental Behaviours*. Internalising and externalising problems* of the child were assessed by caregiver-report on the Child Behaviour Checklist. *CU Traits* were assessed at first assessment by caregiver reports of the Inventory of Callous-Unemotional Traits (ICU). First, to examine group differences in parenting, linear mixed models were computed. Second, to examine the longitudinal associations between parenting and child internalising and externalising problems, random intercept cross-lagged panel models were calculated and it was tested whether the lagged effects differ between groups. At last, multiple regression analyses were computed including group and interaction effects of parenting and CU traits. Parenting behaviour did not significantly differ between the foster and biological family group in both parenting scales. Additionally, the random intercept cross-lagged panel models showed that the lagged effects did not significantly differ between groups. Significant longitudinal cross-lagged effects were found for parental warmth and support and children’s externalising problems across groups. Overall, longitudinal effect sizes were small to medium-sized. Further, CU Traits moderated the relationship between warmth and support and externalising problems of children: in the presence of low CU traits, parental warmth and support and children’s externalising behaviour was negatively associated. No significant interaction effects were found for the other parenting and child problems relationships. The study at hand gives some support for the potential influence of parenting behaviour on internalising and externalising problems in CFC *and* CBF. In particular, parental warmth and support was found to influence children’s behaviour and emotional problems. These findings suggest that parenting behaviours and child psychopathology do influence each other over time reciprocally and to a similar extent in foster and biological families. However, regarding the additional influence of certain child characteristics, such as CU traits, it could be shown that there is a moderation effect on the association between parenting and child development. This might go along with a greater longitudinal stability of some psychopathological symptoms and thereof a reduced responsivity to parental warmth. In summary, the results give hints to tailored parenting behaviours to encourage adaptive developmental pathways in children, that may not always be naturally shown by (foster) parents, but can be promoted in parenting trainings.

## Supplementary Information

Below is the link to the electronic supplementary material.

Supplementary file1 (PDF 28 KB)

## References

[1] Éthier LS, Lemelin J-P, Lacharité C (2004). A longitudinal study of chronic maltreatment on children’s behavioral and emotional problems. Child Abuse Negl.

[2] Oswald S, Heil K, Goldbeck L (2010). History of maltreatment and mental health problems in foster children: a review of the literature. J Pediatr Psychol.

[3] Teicher MH, Andersen SL, Polcari A, Anderson CM, Navalta CP (2002). Developmental neurobiology of childhood stress and trauma. Psychiatr Clin North Am.

[4] Belsky J, Schlomer GL, Ellis BJ (2012). Beyond cumulative risk: distinguishing harshness and unpredictability as determinants of parenting and early life history strategy. Dev Psychol.

[5] McCrory E, Viding E (2015). The theory of latent vulnerability: reconceptualizing the link between childhood maltreatment and psychiatric disorder. Dev Psychopathol.

[6] Goemans A, van Geel M, Vedder P (2015). Over three decades of longitudinal research on the development of foster children: a meta-analysis. Child Abuse Negl.

[7] Orme JG, Buehler C (2001). Foster family characteristics and behavioral and emotional problems of foster children: a narrative review. Fam Relat.

[8] Leve LD, Harold GT, Chamberlain P, Landsverk JA, Fisher PA, Vostanis P (2012). Practitioner review: children in foster care—vulnerabilities and evidence-based interventions that promote resilience processes. J Child Psychol Psychiatry.

[9] Jones R, Everson-Hock ES, Papaioannou D, Guillaume L, Goyder E, Chilcott J (2011). Factors associated with outcomes for looked-after children in young people: a correlates review of the literature. Child: Care. Health Dev.

[10] Rothbaum F, Weisz JR (1994). Parental caregiving and child externalizing behavior in nonclinical samples: a meta-analysis. Psychol Bull.

[11] Yap MBH, Pilkington PD, Ryan SM, Jorm AF (2014). Parental factors associated with depression and anxiety in young people: a systematic review and meta-analysis. J Affect Disord.

[12] Wolfe DA, McIsaac C (2011). Distinguishing between poor/dysfunctional parenting and child emotional treatment. Child Abuse Negl.

[13] Gabler S, Bovenschen I, Lang K, Zimmermann J, Nowacki K, Kliewer J, Spangler G (2014). Foster children’s attachment security and behavior problems in the first six months of placement: associations with foster parents’ stress and sensitivity. Attach Hum Dev.

[14] McLeod BD, Weisz JR, Wood JJ (2007). Examining the association between parenting and childhood depression: a meta-analysis. Clin Psychol Rev.

[15] McLeod BD, Weisz JR, Wood JJ (2007). Examining the association between parenting and childhood anxiety: a meta-analysis. Clin Psychol Rev.

[16] Pinquart M (2017). Associations of parenting dimensions and styles with externalizing problems of children and adolescents: an updated meta-analysis. Dev Psychol.

[17] Vanderfaeillie J, van Holen F, Vanschoonlandt F, Robberechts M, Stroobants T (2013). Children placed in long-term family foster care: a longitudinal study into the development of problem behavior and associated factors. Child Youth Serv Rev.

[18] Chodura S, Lohaus A, Symanzik T, Heinrichs N, Konrad K (2021). Foster parents‘ parenting and the development of children in foster care: a PRISMA-guided literature review and meta-analysis. Clin Child Fam Psychol Rev.

[19] Fisher PA, Gunnar MR, Chamberlain P, Reid JB (2000). Preventive intervention for maltreated preschool children: impact on children's behavior, neuroendocrine activity, and foster parent functioning. J Am Acad Child Adolesc Psychiatry.

[20] Lucey R, Fox RA, Byrnes JB (2006). Maternal characteristics and child problem behavior: a comparison of foster and biological mothers. J Fam Soc Work.

[21] Linares LO, Montalto D, Rosbruch N, Li M (2006). Discipline practices among biological and foster parents. Child Maltreat.

[22] Ehrenberg D, Lohaus A, Konrad K, Heinrichs N (2021) Risikofaktoren und ihre Bedeutung für den Entwicklungsverlauf von Kindern in Pflegeverhältnissen. Submitted

[23] Chodura S, Lohaus A, Symanzik T, Möller C, Heinrichs N, Konrad K (2019). Demografische Eigenschaften von Pflegefamilien in Deutschland. Z Kinder Jugendpsychiatr Psychother.

[24] Belsky J (1984). The determinants of parenting: a process model. Child Dev.

[25] Ellis BJ, Boyce WT, Belsky J, Bakermans-Kranenburg MJ, van Izendoorn MH (2011). Differential susceptibility to the environment: An evolutionary–neurodevelopmental theory. Dev Psychopathol.

[26] Blair RJR, Leibenluft E, Pine DS (2014). Conduct disorder and callous-unemotional traits in youth. N Eng J Med.

[27] Frick PJ, Viding E (2009). Antisocial behavior from a developmental psychopathology perspective. Dev Psychopathol.

[28] Craig SG, Goulter N, Moretti MM (2021). A systematic review of primary and secondary callous-unemotional traits and psychopathy variants in youth. Clin Child Fam Psychol Rev.

[29] Oxford M, Cavell TA, Hughes JN (2003). Callous-unemotional traits moderate the relation between ineffective parenting and child externalizing problems: a partial replication and extension. J Clin Child Adolesc Psychol.

[30] Wootton JM, Frick PJ, Shelton KK, Silverthorn P (1997). Ineffective parenting and childhood conduct problems: the moderating role of callous-unemotional traits. J Consult Clin Psychol.

[31] Pasalich DS, Dadds MR, Hawes DJ, Brennan J (2011). Do callous-unemotional traits moderate the relative importance of parental coercion versus warmth in child conduct problems? An observational study. J Child Psychol Psychiatry.

[32] Kochanska G, Kim S, Boldt LJ, Eun Yoon J (2013). Children’s callous-unemotional traits moderade links between their positive relationships with parents at preschool age and externalizing behavior problems at early school age. J Child Psychol Psychiatry.

[33] Hyde LW, Waller R, Trentacosta CJ, Shaw DS, Neiderhiser JM, Ganiban JM (2016). Heritable and non-heritable pathways to early callous unemotional behavior. Am J Psychiatry.

[34] Waller R, Trentacosta CJ, Shaw DS, Neiderhiser JM, Ganiban JM, Reiss D (2016). Heritable temperament pathways to early callous-unemotional behavior. Br J Psychiatry.

[35] Waller R, Hyde LW, Klump KL, Burt AB (2018). Parenting is an environmental predictor of callous-unemotional traits and aggression: a monozygotic twin differences study. J Am Acad Child Adoles Psychiatry.

[36] Job A-K, Ehrenberg D, Hilpert P, Reindl V, Lohaus A, Konrad K, Heinrichs N (2020). Taking care Triple P for foster parents with young children in foster care: results of a 1-year randomized trial. J Interpers Violence 0886260520909196 10.1177/0886260520909196 32167402

[37] Reitzle M, Winkler Metzke C, Steinhausen H-C (2001). Eltern und Kinder: Der Zürcher Kurzfragebogen zum Erziehungsverhalten (ZKE). Diagnostica.

[38] Achenbach TM, Rescorla L (2000). CBCL/1,5–5 & TRF/1,5–5 profiles.

[39] Achenbach TM (1991). Manual of the Child Behavior Checklist/4–18 and 1991 profile.

[40] Essau CA, Sasagawa S, Frick PJ (2006). Callous-unemotional traits in a community sample of adolescents. Assessment.

[41] Ciucci E, Baroncelli A, Franchi M, Naz Golmaryami F, Frick PJ (2014). The association between callous-unemotional traits and behavioral and academic adjustment in children: further validation of the Inventory of Callous-Unemotional Traits. J Psychopathol Behav Assess.

[42] Winkler J, Stolzenberg H (2009). Adjustierung des Sozialen-Schicht-Index für die Anwendung im Kinder- und Jugendgesundheitssurvey (KiGGS) 2003/2006. Wismarer Diskussionspapiere: 7

[43] R Core Team (2020) A language and environment for statistical computing R Foundation for Statistical Computing. R Core Team¸ Vienna

[44] IBM Corp (2019) IBM SPSS Statistics for Macintosh, Version 26.0. Armonk, NY: IBM Corp

[45] Dong Y, Peng CJ (2013). Principled missing data methods for researchers. Springerplus.

[46] Lüdtke O, Robitzsch A, Trautwein U, Köller O (2007). Umgang mit fehlenden Werten in der psychologischen Forschung. Psychol Rundsch.

[47] van Buuren S, Groothuis-Outsdoorn K (2011). mice: multivariate imputation by chained equations in R. J Stat Softw.

[48] Burns RA, Butterworth P, Kiely KM, Bielak AAM, Luszcz MA, Mitchell P (2011). Multiple imputation was an efficient method for harmonizing the Mini-Mental State Examination with missing item-level data. J Clin Epidemiol.

[49] Lüdecke D (2018). sjmisc: data and variable transformation functions. J Open Source Softw.

[50] Bates D, Maechler M, Bolker B, Walker S (2014). Fitting linear mixed-effects models using lme4. J Stat Softw.

[51] Cnaan A, Laird NM, Slasor P (1998). Using the general mixed model to analyse unbalanced repeated measures and longitudinal data. Stat Med.

[52] Luke SG (2017). Evaluating significance in linear mixed-effects models in R. Behav Res Methods.

[53] Kuznetsova A, Brockhoff PB, Christensen RHB (2017). lmerTest package: tests in linear effects models. J Stat Softw.

[54] Hamaker E, Kuiper RM, Grasman R (2015). A critique of the cross-lagged panel model. Psychol Methods.

[55] Mulder JD, Hamaker EL (2021). Three extensions of the Random Intercept Cross-Lagged Panel Model. Struct Equ Model.

[56] Lohaus A, Kerkhoff D, Chodura S, Möller C, Symanzik T, Rueth JE (2018). Foster children’s mental health problems and parental stress in foster mothers and fathers: longitudinal relationships. Eur J Health Psychol.

[57] Rosseel Y (2012). lavaan: an R package for structural equation modeling. J Stat Softw.

[58] Berkout OV, Gross AM, Young J (2014). Why so many arrows? Introduction to structural equation modeling for the novitiate user. Clin Child Fam Psychol Rev.

[59] Thurber S, Sheehan W (2012). Note on truncated T scores in discrepancy studies with the Child Behavior Checklist and Youth Self Report. Arch Assess Psychol.

[60] Lohaus A, Chodura S, Moeller C, Symanzik T, Ehrenberg D, Job A-K (2017). Children's mental health problems and their relation to parental stress in foster mothers and fathers. Child Adolesc Psychiatry Mental Health.

[61] Fisher PA, Stoolmiller M, Gunnar MR, Burraston BO (2007). Effects of a therapeutic intervention for foster preschoolers on diurnal cortisol activity. Psychoneuroendocrinology.

[62] Reindl V, Schippers A, Tenbrock K, Job A-K, Gerloff C, Lohaus A (2021). Caregiving quality modulates neuroendocrine and immunological markers in young children in foster care who have experienced early adversity. J Child Psychol Psychiatry.

[63] McCrory E, De Brito SA, Viding E (2010). Research review: the neurobiology and genetics of maltreatment and adversity. J Child Psychol Psychiatry.

[64] Pauli R, Tino P, Rogers JC, Baker R, Clanton R, Birch P (2020). Positive and negative parenting in conduct disorder with high versus low levels of callous–unemotional traits. Dev Psychopathol.

[65] Keeble C, Baxter PD, Barber S, Law GR (2015). Participation rates in epidemiology studies and surveys: a review 2007–2015. Int J Epidemiol.

[66] Sugden NA, Moulson MC (2015). Recruitment strategies should not be randomly selected: empirically improving recruitment success and diversity in developmental psychology research. Front Psychol.

