# Supplementary material for: Longitudinal Associations Between Parenting and Child Behaviour Problems and the Moderating Effect of Child Callous Unemotional Traits in Foster and Biological Families
Source: Child Psychiatry Hum Dev. 2022 Mar 3;54(5):1274–86. doi: 10.1007/s10578-022-01324-9 (PMC10435590; doi:10.1007/s10578-022-01324-9)
Supplement: Supplementary file 1 — Supplementary file1 (PDF 28 KB) [file 10578_2022_1324_MOESM1_ESM.pdf]

Supplementary Table 1. Longitudinal Stabilities for Central Variables.

Bivariate Correlation	CFC (N=86)	CBF (N=148)	Fisher's *z*
Warmth / Support T1 to T2	.37**	.58**	*F*=-2.00, *p*=.023
Warmth / Support T1 to T3	.35**	.54**	*F*=-1.73, *p*=.041
Warmth / Support T2 to T3	.59**	.63**	*F*=-.46, *p*=.322
Psychological Pressure, T1 to T2	.52**	.66**	*F*=-1.57, *p*=.058
Psychological Pressure, T1 to T3	.48**	.42**	*F*=.55, *p*=.292
Psychological Pressure, T2 to T3	.70**	.61**	*F*=1.15, *p*=.125
Externalizing Problems, T1 to T2	.83**	.65**	*F*=3.00, *p*=.001
Externalizing Problems, T1 to T3	.77**	.60**	*F*=2.38, *p*=.009
Externalizing Problems, T2 to T3	.84**	.77**	*F*=1.46, *p*=.072
Internalizing Problems, T1 to T2	.70**	.46**	*F*=2.69, *p*=.004
Internalizing Problems, T1 to T3	.73**	.44**	*F*=3.32, *p*<.001
Internalizing Problems, T2 to T3	.72**	.53**	*F*=2.31, *p*=.011

Supplementary Table 2. Intercorrelations between study variables for foster families.

	Ext. Prob., T1	Int. Prob., T1	Ext. Prob., T2	Int. Prob., T2	Ext. Prob., T3	Int. Prob., T3	Warmth/ supp., T1	Psych. Pressure , T1	Warmth/ supp., T2	Psych. Pressure , T2	Warmth/ supp., T3	Psych. Pressure , T3
Ext. Problems, T1												
Int. Problems, T1	.62**											
Ext. Problems, T2	.83**	.56**										
Int. Problems, T2	.51**	.70**	.64**									
Ext. Problems, T3	.77**	.50**	.84**	.53**								
Int. Problems, T3	.43**	.73**	.49**	.72**	.58**							
Warmth/support, T1	-.22	-.18	-.24	-.07	-.24	-.14						
Psych. Pressure, T1	.13	.05	.10	-.04	.15	.11	-.16					
Warmth/support, T2	-.16	-.15	-.14	-.06	-.19	-.20	.37**	.03				
Psych. Pressure , T2	.10	-.10	.11	-.03	.15	.01	-.08	.52**	-.14			
Warmth/support, T3	-.24*	-.12	-.28*	-.08	-.29*	-.10	.35**	-.07	.59**	-.11		
Psych. Pressure , T3	.11	.10	.24	.13	.28*	.24*	-.13	.48**	-.20	.70**	-.36**	

Notes. Pearson correlations were calculated on the imputed data set. *$p < .05$, ** $p < .01$, *** $p < .001$.

Supplementary Table 3. Intercorrelations between study variables for biological families.

	Ext. Prob., T1	Int. Prob., T1	Ext. Prob., T2	Int. Prob., T2	Ext. Prob., T3	Int. Prob., T3	Warmth/ supp., T1	Psych. Pressure , T1	Warmth/ supp., T2	Psych. Pressure , T2	Warmth/ supp., T3	Psych. Pressure , T3
Ext. Problems, T1												
Int. Problems, T1	.66**											
Ext. Problems, T2	.65**	.37**										
Int. Problems, T2	.34**	.46**	.56**									
Ext. Problems, T3	.60**	.24**	.77**	.35**								
Int. Problems, T3	.38**	.44**	.46**	.53**	.55**							
Warmth/support, T1	-.13	-.21*	-.13	-.15	-.09	-.14						
Psych. Pressure, T1	.33**	.30**	.32**	.30**	.24**	.22*	-.44**					
Warmth/support, T2	-.26**	-.22*	-.27**	-.22*	-.26**	-.23**	.58**	-.35**				
Psych. Pressure , T2	.24**	.21*	.30**	.25**	.20*	.06	-.28**	.66**	-.28**			
Warmth/support, T3	-.09	-.10	-.10	-.02	-.14	-.13	.54**	-.17*	.63**	-.15		
Psych. Pressure , T3	.13	.08	.26**	.20*	.22*	.09	-.22*	.42**	-.29**	.61**	-.18*	

Notes. Pearson correlations were calculated on the imputed data set. *$p < .05$, ** $p < .01$, *** $p < .001$.

Supplementary Table 4. Multiple Regression Coefficients on Children's Externalizing Problems.

	Warmth & Support (*SE*)	Psychological Pressure (*SE*)
Intercept	-.11 (.06)	-.13 (.06)*
Group	.27 (.11)*	.34 (.11)**
Parenting Behavior	-.14 (.06)*	.20 (.06)**
CU Traits	.28 (.05)***	.28 (.06)***
Parenting Behavior*CU Traits	-.13 (.06)*	.06 (.06)

Notes. Group: 0 = CBF, 1 = CFC. *$p < .05$, ** $p < .01$, *** $p < .001$. *SE* = Standard Error.

Supplementary Table 5. Multiple Regression Coefficients on Children's Internalizing Problems.

	Warmth & Support (*SE*)	Psychological Pressure (*SE*)
Intercept	-.09 (.06)	-.09 (.07)
Group	.25 (.11)*	.30 (.11)**
Parenting Behavior	-.17 (.06)**	.17 (.06)**
CU Traits	.12 (.06)*	.11 (.06)*
Parenting Behavior*CU Traits	-.04 (.06)	-.06 (.06)

Note. Group: 0 = CBF, 1 = CFC. *$p < .05$, ** $p < .01$, *** $p < .001$. *SE* = Standard Error.
